# New microsatellite markers developed from *Urochloa humidicola *(Poaceae) and cross amplification in different *Urochloa *species

**DOI:** 10.1186/1756-0500-4-523

**Published:** 2011-12-05

**Authors:** Bianca BZ Vigna, Guilherme C Alleoni, Leticia Jungmann, Cacilda B do Valle, Anete P de Souza

**Affiliations:** 1 Center for Molecular Biology and Genetic Engineering (CBMEG), University of Campinas, CP 6010, Campinas, SP CEP 13083-970, Brazil; 2 EMBRAPA Beef Cattle, Plant Biotechnology Laboratory, Brazilian Agricultural Research Corporation, CP 154, Campo Grande, MS CEP 79002-970, Brazil; 3 Department of Plant Biology, Biology Institute, University of Campinas, CP 6109, Campinas, SP CEP 13083-970, Brazil; 4 EMBRAPA Southeastern Region Animal Husbandry, Brazilian Agricultural Research Corporation, CP 339, São Carlos, SP CEP 13560-970, Brazil

## Abstract

**Background:**

*Urochloa humidicola *is a forage grass that grows in tropical regions and is recognized for its tolerance to seasonal flooding. It is a polyploid and apomictic species with high phenotypic plasticity. As molecular tools are important in facilitating the development of new cultivars and in the classification of related species, the objectives of this study were to develop new polymorphic microsatellite markers from an enriched library constructed from *U. humidicola *and to evaluate their transferability to other *Urochloa *species.

**Findings:**

Microsatellite sequences were identified from a previously constructed enriched library, and specific primers were designed for 40 loci. Isolated di-nucleotide repeat motifs were the most abundant followed by tetra-nucleotide repeats. Of the tested loci, 38 displayed polymorphism when screened across 34 polyploid *Urochloa *sp. genotypes, including 20 accessions and six hybrids of *U. humidicola *and two accessions each from *U. brizantha*, *U. dictyoneura*, *U. decumbens *and *U. ruziziensis*. The number of bands per Simple Sequence Repeat (SSR) locus ranged from one to 29 with a mean of 11.5 bands per locus. The mean Polymorphism Information Content (PIC) of all loci was 0.7136, and the mean Discrimination Power (DP) was 0.7873. Six loci amplified in all species tested. STRUCTURE analysis revealed six different allelic pools, and the genetic similarity values analyzed using Jaccard's coefficient ranged from 0.000 to 0.913.

**Conclusions:**

This work reports new polymorphic microsatellite markers that will be useful for breeding programs for *Urochloa humidicola *and other *Urochloa *species as well as for genetic map development, germplasm characterization, evolutionary and taxonomic studies and marker-assisted trait selection.

## Background

*Urochloa humidicola *(Rendle) Morrone & Zuloaga (syn. *Brachiaria humidicola *(Rendle) Schweick..) [1] is an out-crossed and wind-pollinated perennial tropical grass that is widely used for pasture in several tropical regions, including Central and South America, Southeast Asia and Oceania. Also known as koroniviagrass, it is particularly recognized for its tolerance to poorly draining soils, seasonal flooding and infertile acid soils [2], characteristics that led to the successful use of this species in the Amazon region.

*U. humidicola *is a polyploid species that has ploidy levels ranging from tetraploid to heptaploid. The basic chromosome number has been recently reported as *x *= 6 [3-6]. This species reproduces through a *Panicum*-type of apospory [7], which is an asexual mode of reproduction through seeds where somatic cells of the nucellus form unreduced embryo sacs [8].

The difficulty in classifying *Urochloa *grasses is related to subtle differences between species, which are distinguished by slight differences in the small morphological features of the flowers [1,9] and phenotypic plasticity. These subtle differences make the identification of species and intra- and interspecific hybrids problematic and uncertain. As little is known about the genetic makeup of *U. humidicola*, molecular markers represent important tools for elucidating the classification and genetics of the species as well as for applications in breeding programs. More particularly, microsatellite markers are widely used in genetic studies, and due to their high mutation rates [10], they can be especially helpful when comparing closely related individuals [11].

The identification of microsatellite markers depends on knowledge of the flanking region sequences to design appropriate primer pairs. These sequences are usually obtained from enriched libraries [12] or from public sequences. The flanking regions have lower mutation rates than the microsatellites themselves [11] and are often identical among phylogenetically related species, allowing the use of the same markers in these species [13-15].

In a previous study, 384 clones were sequenced and analyzed from a microsatellite-enriched library constructed for *Urochloa humidicola*, and 27 polymorphic microsatellites loci were described [16]. The population structure of the germplasm collection of *U. humidicola *was then analyzed using these loci along with morphological markers [17]. To continue the genetic studies of this species, the present study aimed to develop new microsatellite markers for *U. humidicola*, test their transferability to other *Urochloa *species and validate the newly developed SSRs by evaluating the genetic diversity and population structure among 34 *Urochloa *genotypes (26 of *U. humidicola *and two each of the following species: *U. brizantha*, *U. decumbens*, *U. ruziziensis *and *U. dictyoneura*). The results were compared with previously reported data [17].

## Results

Forty primer pairs were designed and amplified successfully in *U. humidicola*, and 38 SSRs were polymorphic (Table 1). Polymorphism Information Content (PIC) values for each locus were obtained for the 26 *U. humidicola *genotypes as previously described [18]. Discrimination Power (DP) was also determined for each locus [19]. The mean PIC of all loci was 0.7136, and the mean DP was 0.7873. Between one and 29 bands were observed per locus with a mean of 11.5 bands per locus.

**Table 1:** Description of SSR markers developed for Urochloa humidicola (Rendle) Morrone & Zuloaga

						Characteristics in five species*			Characteristics in *U. humidicola*		
						
SSR Locus	Genbank accession number	Repeat Motif	T_a _(°C)	Predicted product size (pb)	Primer Sequences (5'- 3')	N° of bands	Size range (pb)	PIC	N° of bands	PIC	DP
BhUNICAMP028	JF812604	(GT)_4_GG(GT)_3_	57	228	TCTTTTGGCTCTGAATGTGCT TTGATGCCGAATGGAACC	6	195-212	0.7364	5	0.6845	0.9324
BhUNICAMP029	JF812605	(TC)_3_(TG)_8_	55	176	AAGGGATATTTGTGTTTCT TTTTTCAGGATTGCTAAG	7	141-180	0.7363	4	0.6492	0.8708
BhUNICAMP030	JF812606	(AT)_3_TGC(AT)_4_	60	133	GGAATATTGTTGCTGAGAGTGG GCGACGACAGAATAAAAATGAT	3	135-181	0.2211	2	0.1638	0.2123
BhUNICAMP031	JF812607	(GT)_7_	60	126	AGGATTTAAAGGACCCACCAT TCCGCTCGGACTGTGATT	3	126-131	0.3749	3	0.3749	0.6554
BhUNICAMP032	JF812608	(GT)_7_	60	212	GCATATGCAGAGTTTTTGTT TGACCATTTTTCTTATCTTTCT	10	196-222	0.8305	10	0.8305	0.9292
BhUNICAMP033	JF812609	(TG)_5_GG(TG)_3_	60	255	TGAGGTCTTCCGTTCTTGTAGGT ACGAGGCTGCCCGAGTAATC	5	212-285	0.6965	4	0.6356	0.8246
BhUNICAMP034	JF812610	(GA)_20_	51	204	TGTAGTGTTGCTGTAGAGTTA CATTGTTTTGAAGATTTG	3	176-182	0.5894	3	0.5916	0.2800
BhUNICAMP035	JF812611	(AC)_4_(CA)_5_CG(CA)_3_...(CA)_5_	51	248	GATGCATCTCCCTCCCTTACTC AGACATTCATCCCGTTTCACAA	15	160-263	0.8767	13	0.8666	0.9784
BhUNICAMP036	JF812612	(TTG)_9_(TG)_6_TT(TG)_5_CG(TG)_5_	60	265	CGATAGTTAGGCGAGAGTTTTG TATTGTCGTATGGCAGAGTTCA	9	204-282	0.8127	8	0.8052	0.8800
BhUNICAMP037	JF812613	(TG)_8_	55	277	CCGTGGAATCCGACAGGTAG CCGGGAGGAGAGTTAGAAGATG	21	118-302	0.9270	19	0.9238	0.9846
BhUNICAMP038	JF812614	(AG)_13_	60	294	TCTCTTAAGCGACCAGTA CAGGAGATAAGTAAAATGAAT	14	286-321	0.8955	14	0.8975	0.9785
BhUNICAMP039	JF812615	(TC)_9_...(TC)_10_	55	231	CATACTTGCATTCTTTTGAT TGTATGAATTTATTGTTTGACT	22	183-263	0.9252	21	0.9254	0.9938
BhUNICAMP040	JF812616	(CTTG)_3_	60	257	TGTAAGCATATCATTTCGTCTA ACTGCCCTGTTTTCCTG	2	258-261	0.2772	2	0.2784	0.4092
BhUNICAMP041	JF812617	(TC)_3_(AAAAT)_3_	62	178	GCTAGGATGGTGGGCTGTGCT CGACGTTTCCGGAATGATGTTT	6	173-270	0.4797	3	0.3966	0.5662
BhUNICAMP042	JF812618	(TG)_7_	60	223	CCGCTGAGCTGTATAGGAAGTT AAGAGGCGGGACATTTAGGA	6	210-226	0.6465	6	0.6465	0.8400
BhUNICAMP043	JF812619	(GT)_3_(TG)_5_GG(TG)_4_	60	216	TGTGTTTGTGTTCTCTATGTGT TATGTGATCCAAAAGTGAAGTG	5	212-224	0.697	5	0.7367	0.9046
BhUNICAMP044	JF812620	(CA)_11_	60	132	TAACACAAGTGCAGGGCGTAAT TGAACACAGCGACACAAGACAC	17	96-130	0.8913	17	0.8918	0.9815
BhUNICAMP045	JF812621	(AC)_11_	60	245	ACACCACACCAAATTCTTACCC TCTCGTTTCATGGCACTGTCTA	14	225-300	0.8956	14	0.8994	0.9600
BhUNICAMP046	JF812622	(TG)_10_	60	262	ACGCCGCAGCAGTTGGTG TCAGGACGTGCCGATGGTAAT	22	230-284	0.9268	20	0.9192	0.9877
BhUNICAMP047	JF812623	(TC)_20_	57	284	TACATGCAGCAACTAAGATA GCACTAACAAGAAAAGATT	26	211-330	0.9231	23	0.9307	0.9969
BhUNICAMP048	JF812624	(AG)_20_	57	286	GCACTAAACAAGAAAAGATT TACATGCAGCAACTAAGATA	29	212-350	0.8864	23	0.9200	0.9754
BhUNICAMP049	JF812625	(AG)_3_A(AG)_4_	60	285	GGGCCCGGCACAACAGTAG AGGCCCACACGCAGAGAACA	8	184-287	0.5597	2	0.3698	0.4277
BhUNICAMP050	JF812626	(TGCG)_3_	60	236	GTGTGGTTGCAGGACGGATTG TGAGTGCATGACAGGTGACGAA	7	225-263	0.7144	6	0.7037	0.9692
BhUNICAMP051	JF812627	(AC)_17_GC(AC)_6_AT(AG)_7_	60	294	TAGCAATGCATGGATAAGACT TGGAGCTCACCCTAAGAAG	1	297	0	1	0	0
BhUNICAMP052	JF812628	(TG)_5_	60	268	ATAACACGGCCAGAACTA ATGAACAATCGGGGTAT	12	230-280	0.8663	12	0.8622	0.9446
BhUNICAMP053	JF812629	(CA)_12_CG(CA)_9_	60	291	GAGTAAGCTTCTAGGACAGGAT GCTCAAACAACTCGATAATAAC	18	224-320	0.8887	10	0.8603	0.7692
BhUNICAMP054	JF812630	(TG)_9_	60	230	CCATATGTGAAGGCTGCGTGAA GTGGCGGGCTAGTGGCTTATC	15	190-290	0.8262	14	0.8281	0.9385
BhUNICAMP055	JF812631	(TC)_7_	60	261	GGAAAAAGAAAAGCGGACTGAA ACGCAAAATAAATGGCAATGGA	12	240-310	0.8829	10	0.8578	0.9538
BhUNICAMP056	JF812632	(TGTT)_4_	60	239	GCCACAACACGCAAATC ATGTATGAGGACCCAAGTTATG	4	234-249	0.269	4	0.2841	0.2861
BhUNICAMP057	JF812633	(AG)_22_	60	219	AGCGACCTCCAGCAACCTT TTTCCCACTCTTTCCCTCTCAC	21	169-239	0.9091	21	0.9091	0.9846
BhUNICAMP058	JF812634	(TC)_18_	55	279	CTAAACAGGTAAACAGACAAT CAAAACGTGAATACATAACA	9	250-340	0.7913	9	0.7913	0.9015
BhUNICAMP059	JF812635	(ATGT)_3_	55	290	CAATCCATTTTAACAAGAAGTC GCAACAGTCCGTAGTAAGTATC	5	288-305	0.5747	5	0.5783	0.5538
BhUNICAMP060	JF812636	(TTTTG)_3_	55	279	AATCCAAAGTCATCCCCACAAT TTTTTCGGCAACAGACAGGTAA	8	270-290	0.7747	7	0.7721	0.9415
BhUNICAMP061	JF812637	(GT)_14_	60	165	TGATTCAAAACGCCACGATAGG GGACCGGAACACTGCTTACGA	22	147-192	0.9139	19	0.9067	0.9846
BhUNICAMP062	JF812638	(CA)_8_	60	155	CAAACCTCGTGCTCGTG AGATGGGTTCGGCTGTC	18	139-193	0.8919	15	0.8779	0.9631
BhUNICAMP063	JF812639	(GA)_8_G(GA)_14_	60	199	CAAGAAAGCGCGATGAAAAA GAACACAATGGAGAAGCAG GTC	14	173-230	0.8967	14	0.8967	0.9508
BhUNICAMP064	JF812640	(TC)_19_	60	175	CCCCTACTTTTATACGACACAT GAACGAGAGTAGTAGCATTGGT	13	145-180	0.8941	13	0.8941	0.9538
BhUNICAMP065	JF812641	(AATA)_3_	55	198	ATGTCACGTTATCAGCAGAAG GGGCCACATCACCTTTT	1	200	0	1	0	0
BhUNICAMP066	JF812641	(TCTT)_3_	55	218	ATGACAAACTGACCGTATC TAGCAATTTTCTTTATCAACT	10	217-231	0.7489	9	0.7598	0.8923
BhUNICAMP067	JF812642	(CT)_17_...(CT)_5_	60	301	ACCCCCTGTAATTGTTGTCC GATTCAGATGGTTAGCGTGTTA	15	245-335	0.8961	14	0.8941	0.9354

* Species evaluated: Urochloa humidicola (Rendle) Morrone & Zuloaga, Urochloa brizantha (Hochst. ex A. Rich.) R.D. Webster, Urochloa decumbens (Stapf) R.D. Webster, Urochloa dictyoneura (Figure & De Not.) Veldkamp, Urochloa ruziziensis (R. Germ. & C.M. Evrard) Crins

Transferability of the developed SSR primer pairs was tested in two genotypes each of *U. brizantha*, *U. decumbens*, *U. ruziziensis *and *U. dictyoneura *for all the loci under the same PCR conditions used for *U. humidicola*. The number of successfully amplified genotypes per number of genotypes tested per species is shown in Table 2. The following loci did not amplify in any of the tested genotypes of *Urochloa *spp: BhUNICAMP031, BhUNICAMP032, BhUNICAMP042, BhUNICAMP051, BhUNICAMP052, BhUNICAMP057, BhUNICAMP058, BhUNICAMP063 and BhUNICAMP064. Twenty-one loci were amplified in at least one *U. brizantha *genotype, 24 were amplified in *U. decumbens*, six were amplified in *U. ruziziensis*, and 25 were amplified in *U. dictyoneura*.

**Table 2:** Transferability of SSR markers developed for Urochloa humidicola in other Urochloa species

**Transferability**^**a,b**^				
**SSR Locus**	***U. brizantha***	***U. decumbens***	***U. ruziziensis***	***U. dictyoneura***

BhUNICAMP028	2/2	2/2	2/2	2/2

BhUNICAMP029	1/2	1/2	0/2	2/2

BhUNICAMP030	2/2	2/2	2/2	2/2

BhUNICAMP031	0/2	0/2	0/2	0/2

BhUNICAMP032	0/2	0/2	0/2	0/2

BhUNICAMP033	0/2	2/2	0/2	0/2

BhUNICAMP034	2/2	2/2	0/2	2/2

BhUNICAMP035	2/2	2/2	1/2	2/2

BhUNICAMP036	0/2	1/2	0/2	0/2

BhUNICAMP037	2/2	2/2	0/2	2/2

BhUNICAMP038	0/2	1/2	0/2	0/2

BhUNICAMP039	2/2	2/2	0/2	1/2

BhUNICAMP040	2/2	2/2	0/2	0/2

BhUNICAMP041	2/2	1/2	0/2	2/2

BhUNICAMP042	0/2	0/2	0/2	0/2

BhUNICAMP043	0/2	2/2	0/2	0/2

BhUNICAMP044	2/2	2/2	0/2	2/2

BhUNICAMP045	2/2	1/2	0/2	0/2

BhUNICAMP046	2/2	2/2	0/2	2/2

BhUNICAMP047	2/2	1/2	0/2	2/2

BhUNICAMP048	2/2	1/2	0/2	2/2

BhUNICAMP049	2/2	1/2	0/2	1/2

BhUNICAMP050	0/2	0/2	0/2	1/2

BhUNICAMP051	0/2	0/2	0/2	0/2

BhUNICAMP052	0/2	0/2	0/2	0/2

BhUNICAMP053	2/2	2/2	2/2	2/2

BhUNICAMP054	0/2	0/2	0/2	2/2

BhUNICAMP055	2/2	2/2	2/2	2/2

BhUNICAMP056	0/2	0/2	0/2	2/2

BhUNICAMP057	0/2	0/2	0/2	0/2

BhUNICAMP058	0/2	0/2	0/2	0/2

BhUNICAMP059	0/2	0/2	0/2	1/2

BhUNICAMP060	2/2	2/2	0/2	2/2

BhUNICAMP061	2/2	0/2	0/2	2/2

BhUNICAMP062	2/2	2/2	2/2	1/2

BhUNICAMP063	0/2	0/2	0/2	0/2

BhUNICAMP064	0/2	0/2	0/2	0/2

BhUNICAMP065	0/2	0/2	0/2	1/2

BhUNICAMP066	2/2	1/2	0/2	2/2

BhUNICAMP067	0/2	0/2	0/2	1/2

Total	21	24	6	25

a Number of successfully amplified genotypes/Number of tested genotypes

b Nomenclatural classification: Urochloa humidicola (Rendle) Morrone & Zuloaga, Urochloa brizantha (Hochst. ex A. Rich.) R.D. Webster, Urochloa decumbens (Stapf) R.D. Webster, Urochloa dictyoneura (Figure & De Not.) Veldkamp, Urochloa ruziziensis (R. Germ. & C.M. Evrard) Crins

**Figure 1:**
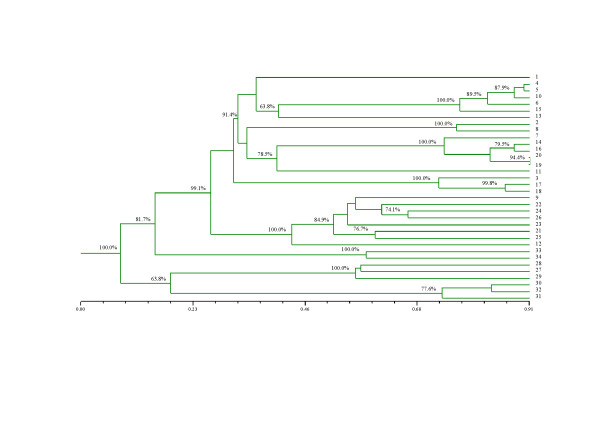
**UPGMA tree representing the relationship among 34 genotypes of the *Urochloa *species using Jaccard's similarity coefficient**. Bootstrap values (*p *< 0.0001) above 60% are indicated, and genotypes are named according to the annotated numbers listed in Table 3.

The genetic similarity values analyzed using Jaccard's coefficient ranged from 0.000 (H125 and H126) to 0.913. See Additional File 1: Genetic similarity based on Jaccard's coefficient. A dendrogram was constructed using the Unweighted Pair-Group Method with the Arithmetic Mean (UPGMA) that successfully discriminated all tested accessions (Figure 1).

**Figure 2:**
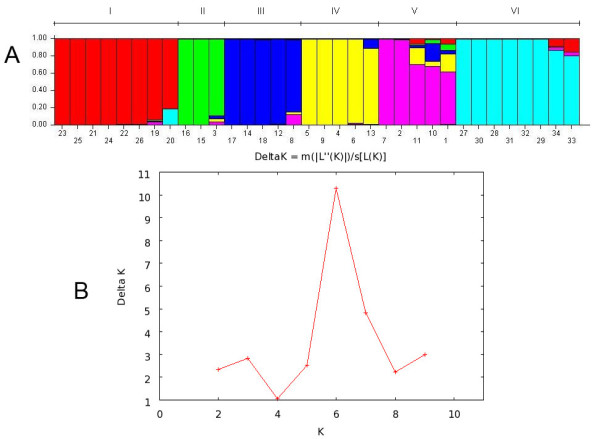
**a Analysis performed with STRUCTURE 2.3 software using an admixture model with correlated allele frequencies**. The clustering profile obtained for *K *= 6 (**b**) is displayed and is indicated by different colors. Each of the 34 genotypes is represented by a single column broken into colored segments with lengths proportional to each of the K inferred gene pools. The left-hand scale indicates the membership coefficients (Q) for allocating genotypes into clusters. Genotypes are named according to the annotated numbers listed in Table 3; four major Clusters of individuals were identified, and Clusters are indicated by numbers I-VI. **b **ΔK values for each K value, calculated according to Evanno et al. [21]. The highest ΔK value corresponds to the optimal K.

The population structure inferred by a model-based Bayesian approach using the STRUCTURE software revealed *K *= 6 clusters. Each cluster was characterized by a set of allele frequencies at each locus and was represented by different colors (red, green, blue, yellow, magenta and light blue) as indicated in Figure 2a. If genotypes indicate admixture, they can be assigned to two or more clusters [20]. We used the term "Cluster" to refer to one or more individuals characterized by a distinguishable allelic set. The best K number of clusters was determined using the ΔK method [21], and its graphical representation is shown in Figure 2b.

In the STRUCTURE analysis, Cluster I (CI-red) was composed of eight genotypes, Cluster II (CII-green) was composed of three genotypes, Cluster III (CIII-blue) was composed of five genotypes, Cluster IV (CIV-yellow) was composed of five genotypes, Cluster V (CV-magenta) was composed of five genotypes and Cluster VI (CVI-light blue) was composed of the last eight genotypes of the studied species. The estimated membership coefficients (Q) of each individual for each allelic pool are shown in Additional File 2: Inferred ancestry of individuals.

## Discussion

A robust set of informative molecular markers for the species of interest is a prerequisite for marker-assisted breeding. *Urochloa humidicola *(or koroniviagrass) is an important tropical forage grass with limited genomic resources. As microsatellite markers are highly polymorphic, reproducible and distributed throughout the genome, they are the ideal marker system for genetic analysis and breeding applications [22]. However, only 27 SSR markers have been reported for koroniviagrass [16]. The present study reports a novel set of SSRs that adds to the existing repertoire of molecular markers in this species and validates the SSRs in some related species.

The majority of the SSRs isolated in the present study were comprised of di-nucleotide repeats (80%) followed by tetra-nucleotide (15%) and penta-nucleotide (5%) repeats. This distribution can be attributed to enrichment of the library for the two di-nucleotide repeats, (AC)_n _and (AG)_n_.

Of all the microsatellites analyzed, 38 out of 40 (95%) showed polymorphism. The most informative loci in this panel of SSRs were the ones with the highest PIC and DP values (BhUNICAMP037, BhUNICAMP039, BhUNICAMP046 and BhUNICAMP047). The BhUNICAMP051 and BhUNICAMP065 loci showed no polymorphism among the studied genotypes, but they may be useful in other studies. The BhUNICAMP030 locus resulted in low PIC and DP values (0.2211 and 0.2123, respectively) as expected because of its low observed polymorphism and its amplification in all other species, which may be a result of a conserved region among the *Urochloa *species studied here.

Of the 40 investigated loci, 18 cross-amplified in at least three other *Urochloa *species, and six cross-amplified in all the evaluated species. The highest success of transferability was obtained in *U. dictyoneura*, where 25 SSR primer pairs were cross-amplified in at least one genotype. These results were expected because the *U. dictyoneura *species is more closely related to *U. humidicola *than to the other three species [9,23]. *U. ruziziensis *was a scoreless species, as only six SSR primer pairs could be cross-amplified. These results are consistent with a previous study with different microsatellite loci [16].

Genetic distance and population structure analysis based on SSR allelic data showed differentiation among *U. humidicola *accessions, hybrids and other *Urochloa *species. Although the number of genotypes is limited, the analyses corroborate a previous study with 60 *U. humidicola *genotypes [17]. The STRUCTURE analysis showed that the genotypes distributed into six major groups, and such groupings were similarly observed by [17]. When examining the dendrogram based on Jaccard's similarity coefficient, the distribution of genotypes was similar to the STRUCTURE analysis, although the two analyses used different statistical approaches.

Indeed, as observed in the amplification profiles, the dendrogram and the allelic pools indicated a closer relationship between *U. humidicola *and *U. dictyoneura *than with the other species.

In the STRUCTURE analysis (Figure 2a), Clusters I-V contained only *U. humidicola *genotypes, and accessions from Clusters II, III and IV were grouped in the same way as has been previously reported [17]. The allelic pools were identified by different colors, and although a genotype might belong to a particular allelic pool, it can also represent a percentage of other allelic pools, as observed in genotypes H016, H31, H006, H013, H012, H044, H035, H030, H004, DT159 and DT157.

Cluster I in the STRUCTURE analysis separated the H016 and H031 accessions (Figure 2a), which were found along with their six hybrids in the same cluster in a previous study [17]. The H031 and H016 accessions were the parents of the first and single mapping population of the species and were the originators of the hybrids used in this study. It is also important to note that these genotypes presented a high degree of divergence, corroborating previous results [17]. Mapping studies are currently underway with the SSR loci reported in this and a previous study [16].

When examining Cluster VI (Figure 2a), which was formed by the other *Urochloa *species, a different allelic pool was found (light blue), and the *U. dictyoneura *accessions (DT159 and DT157) showed some percentage of the red and magenta allelic pools, corroborating what was observed in the dendrogram. When analyzing the grouping pattern of the other *Urochloa *species, it is important to note that only two genotypes of each species were used in contrast to the 26 *U. humidicola *genotypes; this could be biasing the analysis.

As previously observed [17], the H031 accession, which is the sole sexual accession in the germplasm collection, presented a different allelic pool composition. However, when compared to other species, the present study revealed that this accession might have the same origin as the other species based on the high proportion of the blue allelic pool in the genotype.

## Conclusions

The data reported herein indicate that the newly developed SSR markers from *U. humidicola *represent a powerful set of genetic resources for genetic diversity studies and are potentially useful for further studies, including molecular mapping, species and hybrid identification, gene flow and seed purity, in *U. humidicola *and other *Urochloa *species.

## Methods

Thirty-four *Urochloa *genotypes were used in this study. Twenty are *U. humidicola *accessions maintained in the germplasm collection of Embrapa Beef Cattle, six are hybrids from the same species and the other eight are represented by two different accessions from each of the following species: *U. brizantha*, *U. decumbens*, *U. ruziziensis *and *U. dictyoneura*. The annotation numbers, accession numbers (as recorded in Embrapa Beef Cattle (EBC) and Center for Tropical Agriculture (CIAT), genotypes and species identifications are shown in Table 3. Freeze-dried leaf samples were used for DNA extraction following the *cetyl trimethyl ammonium bromide *(CTAB) method previously described [24].

**Table 3:** List of all Urochloa genotypes used in this study

AN	CIAT	BRA	EBC	Genotype	Species
1	16181	4821	H004	germplasm accession	*U. humidicola*

2	16182	4839	H005	germplasm accession	*U. humidicola*

3	16867	4863	H006	germplasm accession	*U. humidicola*

4	16871	4901	H008	germplasm accession	*U. humidicola*

5	16880	4952	H010	germplasm accession	*U. humidicola*

6	16882	4979	H012	germplasm accession	*U. humidicola*

7	16886	5011	H013	germplasm accession	*U. humidicola*

8	26141	5088	H015	germplasm accession	*U. humidicola*

9	26149	5118	H016	germplasm accession	*U. humidicola*

10	16877	4928	H023	germplasm accession	*U. humidicola*

11	16894	5070	H030	germplasm accession	*U. humidicola*

12	26146	5100	H031	germplasm accession	*U. humidicola*

13	26413	6131	H035	germplasm accession	*U. humidicola*

14	26432	6203	H041	germplasm accession	*U. humidicola*

15	16884	4995	H044	germplasm accession	*U. humidicola*

16	NA	NA	H048	germplasm accession	*U. humidicola*

17	NA	1929	H107	germplasm accession	*U. humidicola*

18	6705	2208	H112	germplasm accession	*U. humidicola*

19	6133	1449	H125	germplasm accession	*U. humidicola*

20	6369	0370	H126	germplasm accession	*U. humidicola*

21	-	-	20	hybrid	*U. humidicola*

22	-	-	45	hybrid	*U. humidicola*

23	-	-	184	hybrid	*U. humidicola*

24	-	-	215	hybrid	*U. humidicola*

25	-	-	264	hybrid	*U. humidicola*

26	-	-	320	hybrid	*U. humidicola*

27	16162		B057	germplasm accession	*U. brizantha*

28	16467		B166	germplasm accession	*U. brizantha*

29	16499	004481	D009	germplasm accession	*U. decumbens*

30	26300	004707	D028	germplasm accession	*U. decumbens*

31	26163	005614	R102	germplasm accession	*U. ruziziensis*

32	26174	005614	R104	germplasm accession	*U. ruziziensis*

33	16186	007889	DT157	germplasm accession	*U. dictyoneura*

34	16188	007901	DT159	germplasm accession	*U. dictyoneura*

NA: not available, AN: annotation numbers, CIAT: Center for Tropical Agriculture, BRA and EBC (Embrapa Beef Cattle): codes from each of the Institutions

In a previous study, a microsatellite-enriched library was constructed for *Urochloa humidicola*, and 384 clones were sequenced. The sequences were then treated as described [16], and the microsatellites were identified using the Simple Sequence Repeat Identification Tool (SSRIT) [25]. Only di-nucleotides with five or more repeats, tri-nucleotides with four or more repeats, and tetra-, penta- and hexanucleotides with three or more repeats were considered. Primer pairs were designed using the Primer Select 5.01 (DNASTAR Inc.) and Primer3Plus software [26].

Polymerase chain reactions (PCRs) were carried out as previously described [16]. Amplification products were resolved by electrophoresis in 3% agarose gels prior to vertical electrophoresis in 6% denaturing polyacrylamide gels; gels were then silver stained as previously described [27]. Product sizes were determined by comparison to a 10-bp DNA ladder (Invitrogen, Carlsbad, CA).

The microsatellites were treated as dominant markers due to the polyploid nature of the genotypes. Accordingly, data were scored based on the presence (1) or absence (0) of a band for each of the *Urochloa *genotypes. Both PIC and DP values were calculated to estimate the polymorphism of each locus. PIC values were calculated based on [18] and DP values based on [19]. PIC was used as a tool to measure the information that a given marker locus could provide for the pool of genotypes, whereas DP was used as a quantification tool to measure the efficiency of a given marker for the discrimination of genotypes, i.e., the probability that two randomly chosen individuals have different patterns.

The genetic similarity among all the genotypes was estimated according to Jaccard's similarity coefficient [28] based on a binary matrix constructed with the polymorphic bands. The corresponding genetic similarity matrix was used to generate a dendrogram based on the Unweighted Pair Group Method with the Arithmetic Mean (UPGMA) algorithm as previously reported [29]. All analyses were carried out using NTSYSpc 2.11X [30]. A bootstrap analysis with 10,000 random samplings was applied to estimate the reliability of the dendrogram branches using BOOD version 3.0 [31].

A Bayesian clustering method was employed to assess population structure using the STRUCTURE software version 2.3.3 [20]. We performed 10 runs for each K (from *K *= 1 to *K *= 10) and ran the analysis assuming a model of admixture and correlated allele frequencies. We did not use any prior information about the population origin of the genotypes. A burn-in period of 500,000 generations and MCMC simulations of 700,000 iterations were used in all the above runs. The values of LnP(D) (the log probability of data) were estimated by assigning a prior from 1 to 10, and the optimal K was chosen based on the delta K (ΔK) value [21].

## Competing interests

The authors declare that they have no competing interests.

## Authors' contributions

BBZV carried out computational searches for microsatellite identification, designed flanking primers, participated in microsatellite marker validation, performed the statistical analysis and drafted the manuscript. GCA participated in microsatellite marker validation and statistical analysis. LJ participated in the design and implementation of the study and the microsatellite identification and design of flanking primers. CBV and APS conceived of the study and participated in its design and coordination. APS helped to draft the manuscript. All authors read and approved the final manuscript.

## Supplementary Material

Additional file 1 **Jaccard's similarity coefficients among 34 genotypes of *Urochloa *app evaluated through 40 microsatellite markers**. Individuals are identified according to their EBC code (Table 3) Click here for file

Additional file 2 **The membership coefficient (Q) from STRUCTURE analysis based on 40 microsatellite loci data**. Click here for file
